# Association of Crohn's disease with Foxp3 gene polymorphisms and its colonic expression in Chinese patients

**DOI:** 10.1002/jcla.22835

**Published:** 2019-02-01

**Authors:** Shenglong Xia, Daguan Zhang, Shuzi Zheng, Chaoqun Wu, Qianru Lin, Shijie Ying, Xiaoxiao Shao, Yi Jiang

**Affiliations:** ^1^ Department of Gastroenterology The Second Affiliated Hospital and Yuying Children's Hospital of Wenzhou Medical University Wenzhou China; ^2^ Department of Pediatric Gastroenterology The Second Affiliated Hospital and Yuying Children's Hospital of Wenzhou Medical University Wenzhou China

**Keywords:** Crohn's disease, expression, fork head/winged helix transcription factor, gene polymorphism

## Abstract

**Background:**

Fork head/winged helix transcription factor (Foxp3) plays a pivotal role in regulatory T (Treg) cells. The present study aimed to assess the association of Crohn's disease (CD) with *Foxp3* polymorphisms and its colonic expression in Chinese patients.

**Methods:**

The *Foxp3* polymorphisms, rs3761547, rs2232365, rs2294021, and rs3761548, were examined by *SNaPshot* in 268 CD patients and 490 controls. The colonic expression levels of Foxp3, IL‐2, and IL‐4 were detected in 31 CD patients and 31 controls using real‐time quantitative polymerase chain reaction, immunohistochemistry, and enzyme‐linked immunosorbent assay.

**Results:**

Compared to male controls, the proportion of variant allele of rs3761547 was increased in male patients. The variant alleles of rs3761547, rs2232365, and rs2294021 were less in male patients with stricturing CD compared to those with non‐stricturing, non‐penetrating CD; however, these variants were frequently detected in male patients with colonic CD than in those with ileocolonic CD. The variant allele of rs3761548 was increased in male patients with penetrating CD compared to those with non‐stricturing, non‐penetrating CD. The colonic expression of Foxp3 was higher in CD patients than in controls (both males and females). Compared to male patients carrying wild‐type alleles, the colonic expression of Foxp3 was downregulated in male patients with variant alleles, rs3761547, rs2232365, rs2294021, and rs3761548, respectively. However, the *Foxp3* polymorphisms were not significantly related with the colonic expression levels of IL‐2 and IL‐4 in CD patients (both males and females).

**Conclusion:**

*Foxp3* polymorphisms might increase the CD susceptibility by reducing the colonic expression of Foxp3 in male patients.

## 1 INTRODUCTION

Crohn's disease (CD) is one of the leading clinical phenotypes of inflammatory bowel diseases (IBD). Although the exact etiology is not yet clearly understood, the genetic predisposition and immunological imbalance have been implicated as the cause of the disease.^1^ Regulatory T (Treg) cells and Th17 cells have been described as two distinct subsets of Th1 and Th2 cells due to their opposite effects on autoimmunity. Chao et al^2^ found that Th1 and Th17 subsets were significantly increased in blood circulation, while Treg cells were decreased in Chinese patients with CD as compared to healthy controls. Notably, recent studies have emphasized that the imbalance between Treg cells and Th17 cells is a key process in the intestinal inflammation and the onset of IBD.^3^, ^4^

Fork head/winged helix transcription factor 3 (Foxp3) is primarily expressed in Treg cells and has been demonstrated to program the differentiation and function of these cells. Previous studies have suggested that Foxp3 might switch on a complex transcriptional network responsible for the stabilization of Treg cells’ phenotype. Some studies reported that Treg cells in peripheral blood in patients with active IBD were functionally normal but numerically deficient, and the transcriptional levels of *Foxp3* increased in active IBD lesions as compared to those in non‐inflammatory areas.^5^ Moreover, several observations on murine colitis models deficit in *Foxp3* indicated enhanced vulnerable to inflammation. Conversely, the induction of IBD in mouse models could be blocked by transduction with *Foxp3* into CD4^+^ CD25^−^ Treg cells.^6^ Furthermore, the suppressive function of Treg cells was found to be dependent on the high and stable expression of transcription factor *Foxp3* along with the interaction of Foxp3 with other anti‐inflammatory transcription factors, such as IL‐2 and IL‐4. Moreover, Foxp3 cooperates in a DNA‐binding complex with the nuclear factor of activated T cells that modulate the T‐cell activation and anergy to control the transcription of several key cytokine genes, including *IL‐2*.^7^

The human *Foxp3* is mapped to chromosome Xp11.23, and it encodes the corresponding transcription factor possessing a fork head/winged helix domain, a C2H2 zinc finger domain, and a leucine zipper‐like domain. Several single nucleotide polymorphisms (SNPs) in *Foxp3* affect the expression level of the molecule, thereby resulting in a lack of functional CD4^+^CD25^+^ Treg cells. This deficiency eventually contributes to an increased risk of autoimmune diseases, such as systemic lupus erythematosus, autoimmune thyroid diseases (AITD), and allergic rhinitis (AR).^8^, ^9^, ^10^ Currently, the data from the human genome database indicated that the four SNPs, rs3761547, rs2232365, rs2294021, and rs3761548, are highly frequent in Chinese Han population. Song et al^11^ investigated a cohort of Chinese female population and demonstrated that the major alleles, rs2232365 (T), rs3761547 (A), and rs3761549 (C), contributed to an increased risk of psoriasis vulgaris. Moreover, the mutant homozygotes (AA) of rs3761548 and (CC) of rs2232365 were reported to engender an increasing risk of vitiligo in a Chinese population.^12^ Another study in Chinese population detected *Foxp3* rs2232365, which represented a novel susceptibility locus for unexplained recurrent spontaneous abortion.^13^ The present study aimed to ascertain whether the *Foxp3* polymorphisms rs3761547, rs2232365, rs2294021, and rs3761548 were associated with the predisposition of CD in a Chinese population. In addition, the expression levels of Foxp3, IL‐2, and IL‐4 in colonic tissues were assessed to elucidate the function of *Foxp3* polymorphisms in this cohort of patients with CD.

## 2 MATERIALS AND METHODS

### 2.1 Study subjects

From January 2008 to December 2015, a total of 268 patients with CD were recruited from the Second Affiliated Hospital of Wenzhou Medical University, Zhejiang Province in southeast China. The diagnosis of CD was established based on clinical, endoscopic, radiological, and histopathological findings according to the Lennard‐Jones criteria.^14^ The locations and behaviors of CD were evaluated by the Montreal classification criteria.^15^ Consecutively, a total of 490 age‐ and sex‐matched healthy individuals were assimilated at the Health Examination Center of the Second Affiliated Hospital of Wenzhou Medical University. For all subjects, the exclusion criteria were (a) positive hepatitis markers; (b) diabetes, rheumatoid arthritis, multiple sclerosis, or other autoimmune diseases; (c) acute coronary syndrome or another cardiovascular disease history; (d) various tumors; (e) and IBD family history. The demographic data of CD patients and controls are presented in Table 1.

**Table 1:** Demographic characteristics of patients with Crohn's disease (CD) and the controls

Characteristics	CD	Controls	*P*
Total number	268	490	
Sex (male/female)	137/131	222/268	0.125
Age (means ± SD) (years)	40.61 ± 14.01	41.19 ± 15.75	0.567
Smoking (n [%])			
Current or ex‐smoker	35 (13.06)	74 (15.10)	0.444
Never smoked	233 (86.94)	416 (84.90)	
Body mass index (BMI)	18.18 ± 4.19	21.33 ± 5.21	
Lesion location^a^ (n [%])			
Ileal (L1)	94 (35.07)		
Colonic (L2)	64 (23.88)		
Ileocolonic (L3)	110 (41.05)		
Behavior^a^ (n [%])			
Non‐stricturing, non‐penetrating (B1)	155 (57.84)		
Stricturing(B2)	91 (33.95)		
Penetrating(B3)	22 (8.21)		
Treatment (n [%])			
Sulfasalazine/5‐aminosalicylic acid	212 (79.10)		
Prednisone	143 (53.36)		
Antibiotics	186 (69.40)		
Immunosuppressant	83 (30.97)		
Infliximab	50 (18.66)		
Colectomy	35 (13.06)		

The locations (B1, inflammatory‐disease without evidence of stricture or penetrating disease; B2, stricturing‐constant luminal narrowing with prestenotic dilation and/or obstructive symptoms but without penetrating disease; and B3, penetrating‐bowel perforation, intra‐abdominal fistula, inflammatory mass or abscess not related to a postoperative complication) and behaviors (L1, terminal ileum [TI] without colonic involvement; L2, colonic involvement without TI involvement; L3, ileocolonic disease involving both the TI and colon; and L4, disease proximal to the TI without TI or colonic involvement [coexisting with L1‐L3 disease]) of CD were evaluated by the Montreal classification system. SD, standard deviation.

A subset of participants (31 patients with CD and 31 controls) from each of the two groups (patients with CD and controls) was selected for detecting the expression levels of Foxp3, IL‐2, and IL‐4 in colonic tissue. However, before these measurements, the two subgroups were verified for any significant differences in terms of age or sex. The specimens of inflamed and normal mucosa in the colon were uniformly collected from patients with CD and controls, respectively, during the colonoscopy examination.

The present study was approved by the Local Ethics Committee of Wenzhou Medical University, China, and informed consent was obtained from each patient.

### 2.2 Genomic DNA extraction and genotyping

Genomic DNA was extracted from peripheral blood using the DNeasy Blood & Tissue kit (Qiagen GmbH, Hilden, Germany) and stored at 4°C.

Genotypes of *Foxp3* were determined by multiple *SNaPshot* assays (Applied Biosystems, Foster, CA, USA). Details on the primers used for multiple polymerase chain reaction (PCR) and single‐base extension are listed in Table S1. The multiplex PCR amplification was performed from 10 ng of genomic DNA in a final volume of 10 μL containing 1 μL 10× PCR buffer with MgCl_2_ (15 mmol/L; Roche, Basel, Switzerland), 20 mmol dNTP (Promega, Madison, WI, USA), 0.5 U FastStart *Taq* DNA polymerase (Roche), and a final concentration of each amplification primer of 0.1 μmol/L. The thermal cycler conditions were 95°C for 5 minutes, followed by 35 cycles at 94°C for 30 seconds, 65°C for 30 seconds, and 72°C for 1 minute, with a final incubation at 72°C for 10 minutes. Subsequently, the PCR products were examined by electrophoresis in a 2.5% agarose gel. Then, we purified the PCR products with a mix of 1.5 U shrimp alkaline phosphatase (SAP; New England Biolabs, Beverly, MA, USA) and 2 U exonuclease I (TAKARA, Dalian, China) at 37°C for 80 minutes and enzyme inactivation at 85°C for 15 minutes. The *SNaPshot* multiplex sequencing reactions were performed in a final volume of 7 μL containing 2 μL purified multiple PCR products, 1 μL *SNaPshot* Multiplex Mix, 1 μL 5× sequencing buffer, and 3 μL *SNaPshot* sequencing primers. The cycle conditions were as follows: initial denaturation at 96°C for 1 minute, followed by 25 cycles at 96°C for 10 seconds, 52°C for 5 seconds, and 60°C for 30 seconds. The depuration of the product was carried out by adding 1 U SAP, followed by incubations at 37°C for 60 minutes and 75°C for 15 minutes. Then, 1.5 μL *SNaPshot* products was mixed with 8 μL HiDi™ formamide and 0.5 μL GeneScan‐120LIZ size standard (Applied Biosystems). Sequencing and data analyses were carried out on an ABI 3730 Genetic Analyser with GeneMapper 4.0 software (Applied Biosystems), respectively.

### 2.3 Real‐time quantitative PCR (qPCR) analysis for mRNA expression of *FOXP3*

Total RNA was isolated from intestinal biopsies using the TRIzol™ Reagent (Thermo Fisher Scientific, MA, USA). The cDNA was prepared using the cDNA reverse transcription kit (Thermo Fisher Scientific). The primers were synthesized by the Sangon Biotech (Shanghai, China): 5′ AAGAGCTACGAGCTGCCTGAC 3′ (forward); 5′ GTAGTTTCGTGGATGCCACAG 3′ (reverse) for β‐actin; 5′ GAAACAGCACATTCCAGAGTTC 3′ (forward); 5′ ATGGCCCAGCGGATGAG 3′ (reverse) for Foxp3. qPCR was performed in triplicate, using a Power SYBR Green PCR Master Mix (Applied Biosystems). PCR conditions for gene amplification began with 95°C for 10 minutes, followed by 40 cycles of 95°C for 15 seconds, and 60°C for 1 minute. Amplification was performed in a total volume of 25 μL. The expression of *FOXP3* mRNA was normalized to the expression of β‐actin. Relative gene expression was calculated using the ΔCt method.

### 2.4 Immunohistochemical analysis for the protein expression of *FOXP3*

Biopsy specimens were fixed in formalin and embedded in paraffin. These embedded specimens were cut into 2‐mm sections and boiled in Tris‐EDTA buffer (pH 9.0) in a cooker‐cooler for 25 minutes. After blocking in endogenous peroxidase with 3% hydrogen peroxide solution at room temperature for 25 minutes, the slides were washed in phosphate‐buffered saline (pH 7.4) three times for 5 minutes. The sections were incubated with 3% bovine serum albumin (Solarbio, Beijing, China) for 30 minutes to block unspecific antibody binding. Then, the sections were incubated with mouse anti‐human Foxp3 antibody (Clone 236A/E; Abcam, Cambridge, UK; dilution 1:100) for 24 hours at 4°C and with a secondary antibody (Invitrogen, CA, USA) for 30 minutes in a 37°C water bath. Detection was performed following treatment with immunoperoxidase using the EnVision system (Dako, Glostrup, Denmark). The expression of *Foxp3* was quantitatively evaluated using the Image‐Pro Plus 6.0 analysis system (Media Cybernetics, MD, USA) by calculating the mean density, which was the integrated optical density (IOD) divided by the area of interest. Three fields of each slide at 200× magnification were randomly selected, and the mean density was obtained for further statistical analysis.

### 2.5 ELISA assay

To estimate the levels of IL‐2 and IL‐4 in the colonic tissue, the biopsy samples were homogenized mechanically in the buffer containing 1 mol/L Tris‐HCl, 3 mol/L NaCl, and 10% Triton supplemented with protease inhibitor cocktail (Sigma‐Aldrich). Then, the samples were then centrifugated at 18 300 *g* and 4°C for 30 minutes. The levels of IL‐2 and IL‐4 were examined by ELISA kits according to the manufacturer's instructions (Abcam).

### 2.6 Statistical analysis

Continuous variables are presented as mean ± SD, and categorical variables are described as percentages. The Hardy‐Weinberg equilibrium for the four SNPs in *Foxp3* was evaluated by chi‐square test. Since *Foxp3* is positioned on the X chromosome, both patients with CD and the controls were divided into male and female subgroups. Unconditional logistical regression analysis (backward elimination) was used to investigate the associations of *Foxp3* polymorphisms with the predisposition to CD. The covariants were as follows: age, body mass index (BMI), smoking, drug treatment (sulfasalazine/5‐aminosalicylic acid, prednisone, antibiotics, immunosuppressant, infliximab), and colectomy. Analysis of linkage disequilibrium (LD) and haplotypes across *Foxp3* polymorphisms was performed using Haploview 4.2 software. Post hoc power of the study was estimated using G*Power software (version 3.1). The comparison of the expression levels of Foxp3, IL‐2, and IL‐4 in the colonic tissue was performed using the Student *t* test. Bonferroni correction was applied for multiple comparisons to adjust the statistical threshold (*P *<* *0.05/4 = 0.0125). *P* value < 0.05 was considered significant. Data were analyzed by SPSS 21.0 software for Windows (Chicago, IL, USA).

## 3 RESULTS

### 3.1 Comparison of *Foxp3* polymorphisms between patients with CD and controls

Genotype distributions of *Foxp3* rs3761547, rs2232365, rs2294021, and rs3761548 conformed to the Hardy‐Weinberg equilibrium in controls (all *P *>* *0.05). The frequency of variant allele (G) of rs3761547 was higher in male CD patients than in male controls (29.20% vs 19.82%, odds ratio [OR] = 1.688, 95% confidence interval [CI] = 1.017‐2.735, *P *=* *0.041). After adjusting for age, BMI, smoking, and treatment, the result described above reached a statistical significance (*P *=* *0.013) (Table 2). Nevertheless, no significant association of each *Foxp3* polymorphisms with CD susceptibility was observed in females after the adjustment of these clinical characteristics in patients with CD (all *P *>* *0.05; Table S2).

**Table 2:** Allelic distributions of *Foxp3* gene between patients with Crohn's disease (CD) and controls in males

*Foxp3*	Controls (n = 222)	CD (n = 137)	*P*	OR (95% CI)	*P* ^adjust^	OR^adjust^
rs3761547						
Allele A	178 (80.18)	97 (70.80)	0.041	1.688 (1.017‐2.735)	0.013	2.241 (1.367‐3.675)
Allele G	44 (19.82)	40 (29.20)				
rs2232365						
Allele T	148 (66.67)	78 (56.93)	0.064	1.513 (0.976‐2.345)	0.313	1.122 (0.724‐1.739)
Allele C	74 (33.33)	59 (43.07)				
rs2294021						
Allele A	148 (66.67)	78 (56.93)	0.064	1.513 (0.976‐2.345)	0.189	1.382 (0.891‐2.142)
Allele G	74 (33.33)	59 (43.07)				
rs3761548						
Allele C	191 (86.04)	117 (85.40)	0.867	1.053 (0.574‐1.933)	0.682	1.021 (0.556‐1.874)
Allele A	31 (13.96)	20 (14.60)				

CI, confidence interval; OR, odds ratio.

^adjust^The covariants were as follows: age, body mass index (BMI), smoking, drug treatment (sulfasalazine/5‐aminosalicylic acid, prednisone, antibiotics, immunosuppressant, infliximab), and colectomy.

As described in Table 3, after Bonferroni correction (*P *<* *0.05/4 = 0.0125), the variant alleles (G), (C), (G) of rs3761547, rs2232365, and rs2294021 were shown to be less frequent in male patients with B2‐type CD than in those with B1‐type CD (all *P *<* *0.01). Conversely, in male patients with B1‐type CD, the variant allele (A) of rs3761548 was prevalent in male patients with B3‐type CD (*P *=* *0.001). The variant alleles (G), (C), and (G) of rs3761547, rs2232365, and rs2294021 were more common in male patients with L2‐type than in those with L3‐type (all *P < *0.001). However, no significant differences were observed in the comparisons of *Foxp3* polymorphisms among the subgroups of female patients (all *P *>* *0.0125; Table S3).

**Table 3:** Association of *Foxp3* gene polymorphisms with clinical characteristics of male patients with Crohn's disease (CD)

*Foxp3*	Behavior			Lesion location		
	B1 (n* *=* *74) (%)	B2 (n* *=* *56) (%)	B3 (n* *=* *7) (%)	L1 (n* *=* *45) (%)	L2 (n* *=* *27) (%)	L3 (n* *=* *65) (%)
rs3761547						
Allele A	45 (60.81)	46 (82.14)	6 (85.71)	25 (55.56)	14 (51.85)	58 (89.23)
Allele G	29 (39.19)	10 (17.86)^a^	1 (14.29)	20 (44.44)	13 (48.15)	7 (10.77)^e^
rs2232365						
Allele T	36 (48.65)	41 (73.21)	1 (14.29)	21 (46.67)	8 (29.63)	49 (75.38)
Allele C	38 (51.35)	15 (26.79)^b^	6 (85.71)	24 (53.33)	19 (70.37)	16 (24.62)^f^
rs2294021						
Allele A	36 (48.65)	41 (73.21)	1 (14.29)	21 (46.67)	8 (29.63)	49 (75.38)
Allele G	38 (51.35)	15 (26.79)^c^	6 (85.71)	24 (53.33)	19 (70.37)	16 (24.62)^g^
rs3761548						
Allele C	64 (86.49)	51 (91.07)	2 (28.57)	41 (91.11)	21 (77.78)	55 (84.62)
Allele A	10 (13.51)	5 (8.93)	5 (71.43)^d^	4 (8.89)	6 (22.22)	10 (15.38)

CI, confidence interval; OR, odds ratio. *Bonferroni* correction was used, and the threshold was calculated as 0.05/4 = 0.0125. Non‐Stricturing, non‐penetrating CD vs stricturing CD.

^a^OR = 0.337, 95% CI: 0.147‐0.772, *P* = 0.009, ^b^OR = 0.347; 95% CI: 0.164‐0.731, *P* = 0.005, ^c^OR = 0.347, 95% CI: 0.164‐0.731, *P* = 0.005. Non‐Stricturing, non‐penetrating CD vs penetrating CD, ^d^OR = 16.000, 95% CI: 2.725‐93.941, *P* = 0.001. Colonic CD vs Ileocolonic CD, ^e^OR = 0.130, 95% CI: 0.044‐0.386, *P* < 0.001, ^f^OR = 0.137, 95% CI: 0.051‐0.374, *P* < 0.001, ^g^OR = 0.137, 95% CI: 0.051‐0.374, *P* < 0.001.

**Figure 1:**
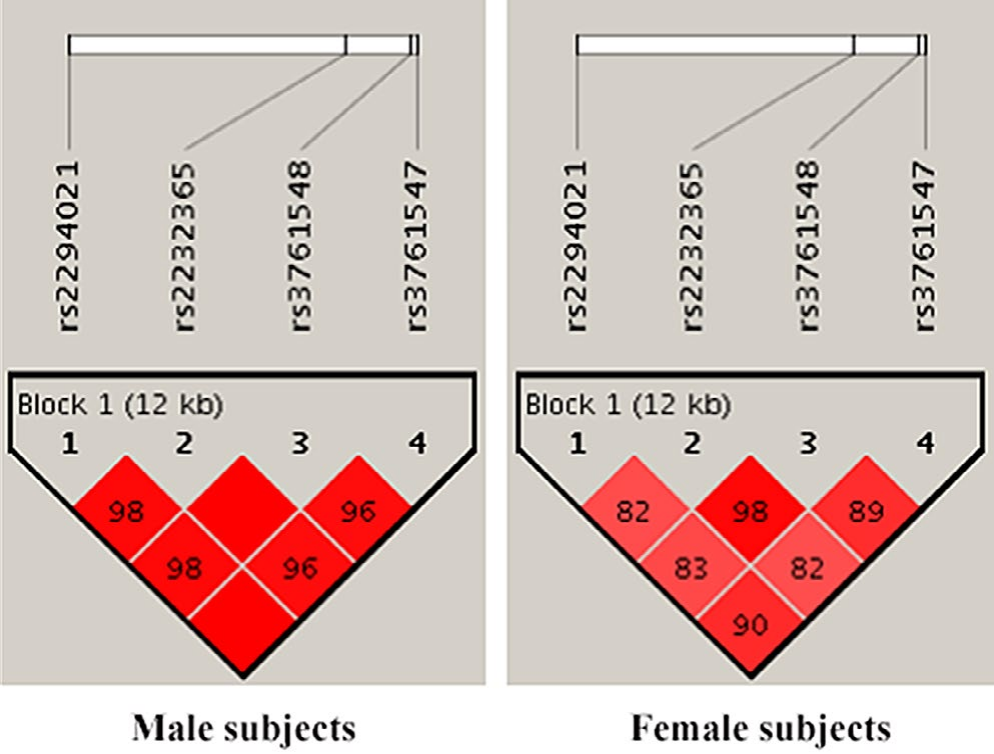
LD patterns between *Foxp3* (rs3761547, rs2232365, rs2294021, rs3761548) polymorphisms by Haploview 4.2 software. Number inside each square represents for *D*′ value between two SNPs and the dark area which have no digital represent *D*′ = 1. Dark color of a square indicates strong connection between the two SNPs. Block 1 illustrated a strong LD among the four SNPs of *Foxp3*

As illustrated in Figure 1, the male and female study subjects showed a strong LD between the SNPs rs3761547, rs2232365, rs2294021, and rs3761548 in *Foxp3*. Furthermore, we analyzed the associations between the haplotypes with CD susceptibility and the clinicopathological characteristics of CD patients. The frequencies of haplotypes due to the four SNPs are summarized in Table 4. Among the seven haplotypes, only (ATAC), (GCGC), and (ACGA) were evaluated, since their frequencies were more than 3% in both CD patients and the controls. When compared to the corresponding controls, the haplotype (GCGC) was more prevalent in male CD patients (*P *=* *0.032), whereas the haplotype (ACGA) was less frequent in female CD patients (*P *=* *0.046). The proportion of haplotype (ATAC) was significantly higher in male patients with B2‐type than in male patients with B1‐type (*P *=* *0.003). However, a converse conclusion was drawn for the haplotype (GCGC) when the above two subgroups were compared (*P *=* *0.009). The haplotype (ACGA) was more prevalent in male patients with B3‐type than in males with B1‐type (*P *<* *0.001). In contrast to male patients with L3‐type, the haplotype (ATAC) was remarkably reduced in the male L2‐type subgroup (*P *<* *0.001), while the haplotype (GCGC) was apparently increased in the male L2‐type subgroup (*P *<* *0.001).

**Table 4:** Haplotype frequencies of *Foxp3* (rs3761547, rs2232365, rs2294021, rs3761548) in patients with Crohn's disease (CD) and controls

Haplotypes	ATAC	ACGA	GCGC
Male controls (n* *=* *222) (%)	147 (66.22)	31 (13.96)	43 (19.37)
Male patients with CD (n* *=* *137) (%)	77 (56.20)	19 (13.87)	40 (29.20)^a^
B1 (n* *=* *74) (%)	35 (47.30)	9 (12.16)	29 (39.19)
B2 (n* *=* *56) (%)	41 (73.21)^b^	5 (8.93)	10 (17.86)^c^
B3 (n* *=* *7) (%)	1 (14.29)	5 (71.43)^d^	1 (14.29)
L1 (n* *=* *45) (%)	21 (46.67)	4 (8.89)	20 (44.44)
L2 (n* *=* *27) (%)	7 (25.93)^e^	5 (18.52)	14 (51.85)^f^
L3 (n* *=* *65) (%)	49 (75.38)	10 (15.38)	6 (9.23)
Female controls (2n = 536) (%)	301 (56.16)	102 (19.03)	100 (18.66)
Female patients with CD (2n = 262) (%)	158 (60.31)	35 (13.36)^g^	60 (22.90)
B1 (2n = 162) (%)	98 (60.49)	22 (13.58)	36 (22.22)
B2 (2n = 70) (%)	48 (68.57)	5 (7.14)	14 (20.00)
B3 (2n = 30) (%)	12 (40.00)	8 (26.67)	10 (33.33)
L1 (2n = 98) (%)	55 (56.12)	10 (10.20)	30 (30.61)
L2 (2n = 74) (%)	40 (54.05)	12 (16.22)	17 (22.97)
L3 (2n = 90) (%)	63 (70.00)	13 (14.44)	13 (14.44)

CI, confidence interval; OR, odds ratio. *Bonferroni* correction was particularly used for stratified analyses, and the threshold was calculated as 0.05/4 = 0.0125. Haplotype frequencies less than 3% were not shown in the table.

^a^Male patients with CD vs Controls, OR = 1.717, 95% CI: 1.045‐2.820, *P* = 0.032. Non‐Stricturing, non‐penetrating CD vs stricturing CD, ^b^OR = 0.328, 95% CI: 0.156‐0.693, *P* = 0.003, ^c^OR = 2.946, 95% CI: 1.295‐6.784, *P* = 0.009. Non‐Stricturing, non‐penetrating CD vs penetrating CD, ^d^OR = 0.055, 95% CI: 0.009‐0.329, *P* < 0.001. Colonic CD vs Ileocolonic CD, ^e^OR = 0.114, 95% CI: 0.041‐0.320, *P* < 0.001, ^f^OR = 10.590, 95% CI: 3.423‐32.758, *P* < 0.001. ^g^Female patients with CD vs Controls, OR = 0.656, 95% CI: 0.433‐0.995, *P* = 0.046.

### 3.2 Correlation between *Foxp3* polymorphisms and the colonic expression levels in patients with CD

**Figure 2:**
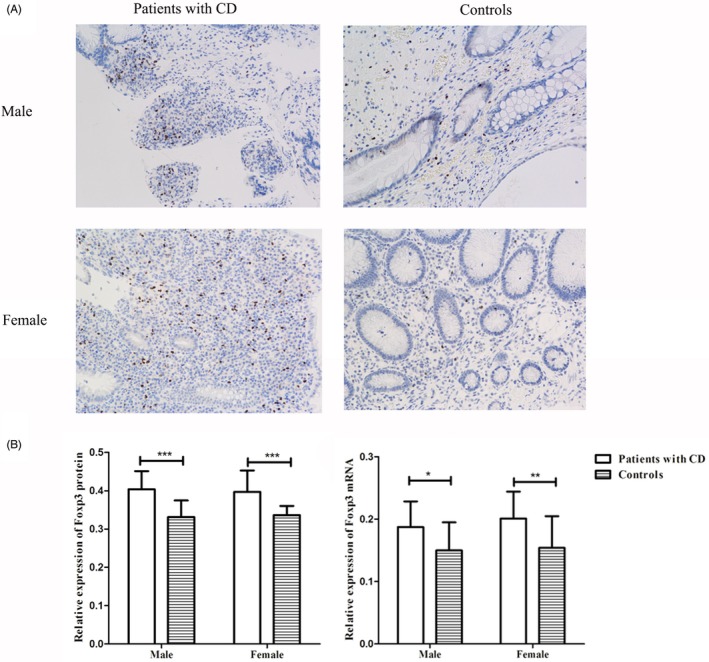
Expression of *Foxp3* in colonic tissues from patients with CD and controls. The protein expression and mRNA expression of *FOXP3* were evaluated quantitatively using the Image‐Pro Plus 6.0 and the ΔCt method, respectively. A, Immunohistological images of the expression of Foxp3. Foxp3 was detected in mucosa lamina propria with nuclear staining. Sections are shown at magnifications of ×200. B, The protein expression and mRNA expression of *Foxp3* in male and female subjects. *P <* *0.05, **P <* *0.01, ***P <* *0.001

**Figure 3:**
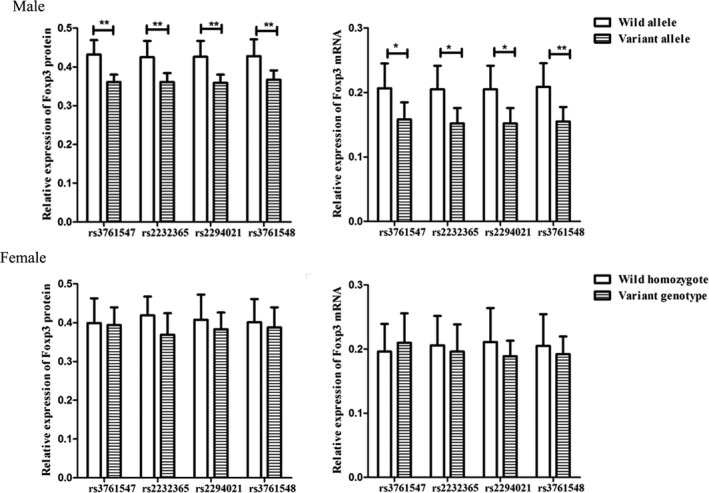
Expression of *Foxp3* protein and mRNA in patients with CD harboring the different alleles or genotypes of *Foxp3* (rs3761547, rs2232365, rs2294021, and rs376154). The protein expression and mRNA expression of *Foxp3* were quantitatively evaluated using the Image‐Pro Plus 6.0 and the ΔCt method, respectively. Variant genotypes: heterozygote and variant homozygote. *P <* *0.05, **P <* *0.01, ***P <* *0.001

The average expression of Foxp3 mRNA and protein was upregulated in patients with CD as compared to controls (in males or females; Figure 2). Moreover, the average expression of Foxp3 mRNA and protein in male patients with CD carrying the variant allele (G), (C), (G), and (A) was downregulated as compared to that in male patients with the wild‐type allele (A), (T), (A), and (C) of rs3761547, rs2232365, rs2294021, and rs3761548, respectively. However, no significant difference was detected between the variant genotypes and wild‐type homozygotes of the four SNPs in female patients with CD (all *P *>* *0.05; Figure 3).

### 3.3 Association between *Foxp3* polymorphisms and the expression levels of IL‐2 and IL‐4 in patients with CD

**Figure 4:**
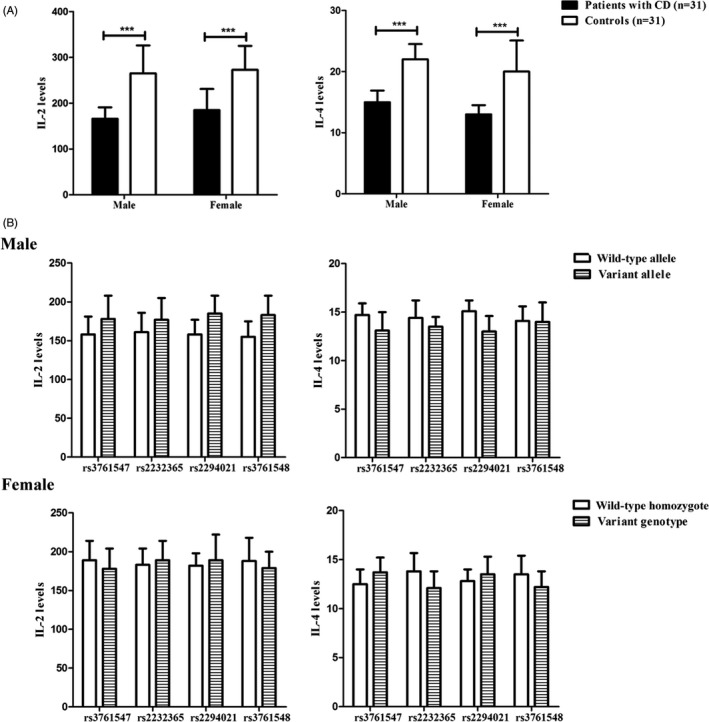
Expression levels of IL‐2 and IL‐4 in colonic tissues from patients with CD and controls (A). The association of *Foxp3* polymorphisms with expression levels of IL‐2 and IL‐4 in male and female patients with CD (B). Variant genotypes: heterozygote and variant homozygote. *P <* *0.05, **P <* *0.01, ***P <* *0.001

Previous studies reported that Foxp3 might be responsible for the modulation of several key cytokines in T‐cell activation and anergy, such as IL‐2 and IL‐4.^7^ Therefore, we further detected the colonic expression levels of the two cytokines by ELISA. The colonic expressions of IL‐2 and IL‐4 in patients with CD were lower than that in controls (both males and females). Nevertheless, no distinct correlation was established between the four SNPs and the expression levels of IL‐2 and IL‐4 in this cohort of patients with CD (in males and females; all *P *>* *0.05; Figure 4).

## 4 DISCUSSION

Evidence from *Foxp3*‐mutant scurfy mice and *Foxp3*‐null mice has indicated that both were likely to suffer from autoimmune and inflammatory syndromes, mainly due to the deficiency of CD4^+^CD25^+^ Treg cells.^16^, ^17^ Conversely, the retroviral gene transfer of *Foxp3* into neonatal *Foxp3*‐deficient mice successfully induced the differentiation of naive T cells into a Treg cell phenotype.^16^, ^17^, ^18^ Moreover, the dysfunction of *Foxp3* might not only give rise to the lack of Treg cells but also promote the activity of CD4^+^ CD25^−^ T cells, which expressed the increased levels of several inflammatory markers and cytokines, leading to autoimmune diseases. Several SNPs of *Foxp3* that occur at high frequencies in the general population have been widely investigated in common multifactorial human diseases, such as AITD, immunodysregulation, polyendocrinopathy, enteropathy, X‐linked (IPEX) syndrome, and type 1 diabetes.^19^, ^20^

The present study primarily suggested that the rs3761547 variation might confer an enhanced risk for CD in male individuals. Moreover, the rs3761547, rs2232365, rs2294021, and rs3761548 variations, as well as the haplotypes GCGC, ACGA, and ATAC formed by the four SNPs, might exert a potential effect on the behaviors and/or locations of CD in male patients. These findings suggested that *Foxp3* might be a candidate gene for CD in Chinese males. Several genetic studies implicated the polymorphisms of *Foxp3* in the risk of autoimmune and allergic diseases and demonstrated significant gender‐based differences in the distribution of *Foxp3* polymorphisms. For instance, a previous study in Hungarian population showed that the variant homozygote (AA) of rs3761548 was a protective factor against AR in female patients.^9^ However, the allele and genotype distributions of this SNP did not differ significantly between the male patients and controls.^9^ Gao et al investigated a Chinese Han population and reported that the variant genotype (CA+AA) of *Foxp3* rs3761548 engendered the enhanced risk of psoriasis in male individuals. The study speculated that this gender discrepancy was primarily attributed to the localization of *Foxp3* on the X chromosome.^21^

Wildin et al^22^ found that the genes on the alternative X chromosome could interact with *Foxp3* locus via X inactivation, thereby modifying the expression of *Foxp3*. Moreover, the effects of gender‐specific hormones (eg, estrogen) or imprinting and an epigenetic phenomenon, wherein the allelic expression relies on the gender of the parent from whom this specific allele is inherited, cannot be ignored.^22^, ^23^ Interestingly, the Y chromosome‐linked polymorphisms might differentially regulate the expression of X‐linked genes.^24^ Thus, it can be hypothesized that the Foxp3 gene expression might be differentially modulated in males and females through the suppressive or activation mechanisms mediated by the Y chromosome.

Subsequently, the present study analyzed the influence of *Foxp3* polymorphisms on the colonic expression of Foxp3 in patients with CD. Consistent with the results of the previous study by Wang et al,^25^ the colonic expressions of *Foxp3* mRNA and protein are shown to be upregulated in patients with CD. Moreover, the variations of rs3761547, rs2232365, rs2294021, and rs3761548 were related to the low colonic expressions of Foxp3 in male patients but not in females. Theoretically, rs3761547, rs2232365, and rs3761548 occur in the promoter region of *Foxp3* that could foster a decreased expression of Foxp3 by preventing the binding of some transcription factors to the corresponding site.^26^, ^27^, ^28^ In addition, *Foxp3* rs2294021 exists in the 3′‐untranslated intron region of the gene and modulates the *Foxp3* mRNA expression level as well as the binding of *Foxp3* mRNA to other proteins.^29^ Furthermore, in a related biochemical assay, the minor allele of rs2294021 was reported to enhance the *Foxp3* mRNA expression owing to the dramatically improved transcriptional activity of *Foxp3*.^29^ Another previous study from Denmark reported that the rs3761548 variation might elevate the risk of CD due to skewed X chromosome inactivation (SXCI) in male individuals as opposed to females; the population differences were characterized by low expression levels of *Foxp3* and the decreased numbers of Treg cells.^30^ Moreover, the above studies explained the genetic variations of *Foxp3* might contribute to the increased risk of CD in males.

Notably, in the current study, the females carrying haplotype (ACGA) were less susceptible to CD. However, we failed to draw similar conclusions when the four SNPs in the Foxp3 gene among female CD patients and the controls were investigated. To date, none of the studies have associated the haplotypes of *Foxp3* polymorphisms to CD susceptibility or its clinical outcome, thereby making the present study as the first to describe such a correlation.

In summary, the present study suggested that *Foxp3* polymorphisms might increase the CD susceptibility by reducing the colonic expression of Foxp3 in males. Nevertheless, the colonic expression of Foxp3 was not measured in all patients, but only in subgroups. Nonetheless, whether the subgroups used for examining the expression of Foxp3 were representative of the full cohort of study subjects in terms of the influences of gender, *Foxp3* polymorphisms, or predisposition of CD in patients could not be confirmed. In addition, the present study on the association of *Foxp3* polymorphisms with the risk of CD had a poor statistical power (<80% for each of the SNPs) due to limited sample size. Thus, large sample studies or population replication studies are imperative for identifying the precise relationship between *Foxp3* polymorphisms and CD susceptibility.

## CONFLICT OF INTEREST

The authors declare that they have no competing interests.
