# Supplementary material for: Association of Crohn's disease with Foxp3 gene polymorphisms and its colonic expression in Chinese patients
Source: J Clin Lab Anal. 2019 Feb 1;33(4):e22835. doi: 10.1002/jcla.22835 (PMC6528575; doi:10.1002/jcla.22835)
Supplement: Supplementary file 1 [file JCLA-33-e22835-s001.doc]

**Supplementary Table 1**. Amplification and extension primers of Foxp3 gene.

| SNP | Amplification Primers(5'→3') | Size (bp) | Extension Primers(5'→3') | Size (bp) |
| --- | --- | --- | --- | --- |
| rs2232365 | F: GAGGCGAGTCCAGGAGTGTGAT  R: AGAGGAGAAGGAGTGGGCATTTGA | 194 | **24T**-GTGACAGAGAGGAGGAGAGA | 44 |
| rs2294021 | F: GGTACACATGAGGACCCTCCACTG  R: CCCAGCCAGCCAATTAGCAGATG | 249 | **27T**-GATCTGGCAGACACCATGGC | 47 |
| rs3761547 | F: CCAACGTGTGAGAAGGCAGAAGG  R: GCAGCGGCAGAGTTGAAATCCA | 160 | **21T**-GTCCCCTGGATAGAGGGGCA | 41 |
| rs3761548 | F: TCTCTGGTCTTCAATTTGCCCTTCTAC  R: CTCTCTTGCTCGCTCTTTGTGTGT | 202 | **35T**-TCTGGCTCTCTCCCCAACTG | 55 |

Bold characters refer to the 5' poly-thymidine tail.
