# Supplementary material for: Association of Crohn's disease with Foxp3 gene polymorphisms and its colonic expression in Chinese patients
Source: J Clin Lab Anal. 2019 Feb 1;33(4):e22835. doi: 10.1002/jcla.22835 (PMC6528575; doi:10.1002/jcla.22835)
Supplement: Supplementary file 2 [file JCLA-33-e22835-s002.doc]

**Supplementary Table 2**. Allelic and genotypic distributions of *Foxp3* between patients with Crohn's disease (CD) and controls in females.

| *Foxp3* | Controls (n=268) | CD (n=131) | *P* | *OR*(95%*CI*) | *P^adjust^* | *OR^adjust^* |
| --- | --- | --- | --- | --- | --- | --- |
| rs3761547 |  |  |  |  |  |  |
| AA | 179  (66.79) | 83  (63.36) |  |  |  |  |
| AG | 62  (23.13) | 30  (22.90) |  |  |  |  |
| GG | 27  (10.08) | 18  (13.74) |  |  |  |  |
| AG+GG | 89  (33.21) | 48  (36.64) | 0.498 | 1.163  (0.751-1.801) | 0.551 | 1.262  (0.815-1.954) |
| Allele A | 420  (78.36) | 196  (74.81) | 0.262 | 1.219  (0.862-1.724) | 0.311 | 1.321  (0.934-1.868) |
| Allele G | 116  (21.64) | 66  (25.19) |
| rs2232365 |  |  |  |  |  |  |
| TT | 110  (41.04) | 59  (45.04) |  |  |  |  |
| TC | 108  (40.30) | 46  (35.11) |  |  |  |  |
| CC | 50  (18.66) | 26  (19.85) |  |  |  |  |
| TC+CC | 158  (58.96) | 72  (54.96) | 0.448 | 0.850  (0.557-1.295) | 0.394 | 0.669  (0.439-1.019) |
| Allele T | 328  (61.19) | 164  (62.60) | 0.702 | 0.942  (0.695-1.278) | 0.631 | 0.881  (0.650-1.195) |
| Allele C | 208  (38.81) | 98  (37.40) |
| rs2294021 |  |  |  |  |  |  |
| AA | 108  (40.30) | 58  (44.28) |  |  |  |  |
| AG | 105  (39.18) | 48  (36.64) |  |  |  |  |
| GG | 55  (20.52) | 25  (19.08) |  |  |  |  |
| AG+GG | 160  (59.70) | 73  (55.72) | 0.449 | 0.850  (0.557-1.296) | 0.512 | 0.827  (0.542-1.261) |
| Allele A | 321  (59.89) | 164  (62.60) | 0.462 | 0.892  (0.658-1.209) | 0.537 | 0.910  (0.671-1.233) |
| Allele G | 215  (40.11) | 98  (37.40) |
| rs3761548 |  |  |  |  |  |  |
| CC | 173  (64.55) | 100  (76.34) |  |  |  |  |
| CA | 73  (27.24) | 25  (19.08) |  |  |  |  |
| AA | 22  (8.21) | 6  (4.58) |  |  |  |  |
| CA+AA | 95  (35.45) | 31  (23.66) | 0.017 | 0.565  (0.351-0.907) | 0.061 | 1.559  (0.970-2.505) |
| Allele C | 419  (78.17) | 225  (85.88) | 0.010 | 0.589  (0.393-0.882) | 0.056 | 1.442  (0.963-2.519) |
| Allele A | 117  (21.83) | 37  (14.12) |

*^adjust^* The covariants were as follows: age, body mass index (BMI), smoking, drug treatment (sulfasalazine/5-aminosalicylic acid, prednisone, antibiotics, immunosuppressant, infliximab) and colectomy. *OR*: odds ratio. *CI*: confidence interval.
