# Supplementary material for: Association of Crohn's disease with Foxp3 gene polymorphisms and its colonic expression in Chinese patients
Source: J Clin Lab Anal. 2019 Feb 1;33(4):e22835. doi: 10.1002/jcla.22835 (PMC6528575; doi:10.1002/jcla.22835)
Supplement: Supplementary file 3 [file JCLA-33-e22835-s003.doc]

**Supplementary Table 3**. Association of *Foxp3* gene polymorphisms with clinical characteristics of female patients with Crohn's disease (CD).

| Foxp3 gene | Behavior | | |  | Lesion location | | |
| --- | --- | --- | --- | --- | --- | --- | --- |
|  | B1(*n*=81) (%) | B2 (*n*=35) (%) | B3(*n*=15) (%) |  | L1 (*n*=49) (%) | L2 (*n*=37) (%) | L3 (*n*=45) (%) |
| rs3761547 |  |  |  |  |  |  |  |
| AA | 54(66.67) | 21(60.00) | 8(53.33) |  | 29(59.18) | 21(56.76) | 33(73.33) |
| AG+GG | 27(33.33) | 14(40.00) | 7(46.67) |  | 20(40.82) | 16(43.24) | 12(26.67) |
| Allele A | 122(75.31) | 54(77.14) | 20(66.67) |  | 68(69.39) | 52(70.27) | 76(84.44) |
| Allele G | 40(24.69) | 16(22.86) | 10(33.33) |  | 30(30.61) | 22(29.73) | 14(15.56) |
| rs2232365 |  |  |  |  |  |  |  |
| TT | 37(45.68) | 18(51.43) | 4(26.67) |  | 24(48.98) | 11(29.73) | 24(53.33) |
| TC+CC | 44(54.32) | 17(48.57) | 11(73.33) |  | 25(51.02) | 26(70.27) | 21(46.67) |
| Allele T | 99(61.11) | 51(72.86) | 14(46.67) |  | 56(57.14) | 44(59.46) | 64(71.11) |
| Allele C | 63(38.89) | 19(27.14) | 16(53.33) |  | 42(42.86) | 30(40.54) | 26(28.89) |
| rs2294021 |  |  |  |  |  |  |  |
| AA | 37(45.68) | 17(48.57) | 4(26.67) |  | 24(48.98) | 10(27.03) | 24(53.33) |
| AG+GG | 44(54.32) | 18(51.43) | 11(73.33) |  | 25(51.02) | 27(72.97) | 21(46.67) |
| Allele A | 100(61.25) | 51(72.86) | 14(46.67) |  | 59(60.20) | 43(58.11) | 62(68.89) |
| Allele G | 62(38.75) | 19(27.14) | 16(53.33) |  | 39(39.80) | 31(41.89) | 28(31.11) |
| rs3761548 |  |  |  |  |  |  |  |
| CC | 62(75.31) | 29(82.86) | 9(60.00) |  | 41(83.67) | 25(67.57) | 34(75.56) |
| CA+AA | 19(24.69) | 6(17.14) | 6(40.00) |  | 8(16.33) | 12(32.43) | 11(24.44) |
| Allele C | 139(85.80) | 64(91.43) | 22(73.33) |  | 87(88.78) | 61(82.43) | 77(85.56) |
| Allele A | 54(66.67) | 21(60.00) | 8(53.33) |  | 11(11.22) | 13(17.57) | 13(14.44) |

*OR*: odds ratio. *CI*: confidence interval. *Bonferroni* correction was used and the threshold was calculated as 0.05/4=0.0125.
